# Improving HIV Outgrowth by Optimizing Cell-Culture Conditions and Supplementing With *all-trans* Retinoic Acid

**DOI:** 10.3389/fmicb.2020.00902

**Published:** 2020-05-15

**Authors:** Yuwei Zhang, Delphine Planas, Laurence Raymond Marchand, Marta Massanella, Huicheng Chen, Vanessa Sue Wacleche, Annie Gosselin, Jean-Philippe Goulet, Mario Filion, Jean-Pierre Routy, Nicolas Chomont, Petronela Ancuta

**Affiliations:** ^1^Département de microbiologie, infectiologie et immunologie, Faculté de Médecine, Université de Montréal, Montreal, QC, Canada; ^2^Centre hospitalier de l’Université de Montréal (CHUM)-Research Centre, Montreal, QC, Canada; ^3^Caprion, Montreal, QC, Canada; ^4^Alethia Biotherapeutics, Montreal, QC, Canada; ^5^McGill University Health Centre, Montreal, QC, Canada

**Keywords:** memory CD4^+^ T-cells, ART, HIV-1 reservoirs, ATRA, QVOAs

## Abstract

The persistence of replication-competent HIV reservoirs in people living with HIV (PLWH) receiving antiretroviral therapy (ART) is a barrier to cure. Therefore, their accurate quantification is essential for evaluating the efficacy of new therapeutic interventions and orienting the decision to interrupt ART. Quantitative viral outgrowth assays (QVOAs) represent the “*gold standard*” for measuring the size of replication-competent HIV reservoirs. However, they require large numbers of cells and are technically challenging. This justifies the need for the development of novel simplified methods adapted for small biological samples. Herein, we sought to simplify the viral outgrowth procedure (VOP) by (*i*) using memory CD4^+^ T-cells, documented to be enriched in HIV reservoirs (*ii*) optimizing cell-culture conditions, and (*iii*) supplementing with a*ll-trans* retinoic acid (ATRA), a positive regulator of HIV replication. Memory CD4^+^ T-cells were sorted from the peripheral blood of ART-treated (HIV+ART; *n* = 14) and untreated (HIV+; *n* = 5) PLWH. The VOP was first performed with one original replicate of 1 × 10^6^ cells/well in 48-well plates. Cells were stimulated *via* CD3/CD28 for 3 days, washed to remove residual CD3/CD28 Abs, split every 3 days for optimal cell density, and cultured in the presence or the absence of ATRA for 12 days. Soluble and intracellular HIV-p24 levels were quantified by ELISA and flow cytometry, respectively. Optimal cell-culture density achieved by splitting improved HIV outgrowth detection. ATRA promoted superior/accelerated detection of replication-competent HIV in all HIV+ART individuals tested, including those with low/undetectable viral outgrowth in the absence of ATRA. Finally, this VOP was used to design a simplified ATRA-based QVOA by including 4 and 6 original replicates of 1 × 10^6^ cells/well in 48-well plates and 2 × 10^5^ cells/well in 96-well plates, respectively. Consistently, the number of infectious units per million cells (IUPM) was significantly increased in the presence of ATRA. In conclusion, we demonstrate that memory CD4^+^ T-cell splitting for optimal density in culture and ATRA supplementation significantly improved the efficacy of HIV outgrowth in a simplified ATRA-based QVOA performed in the absence of feeder/target cells or indicator cell lines.

## Introduction

Antiretroviral therapy (ART) significantly reduces the morbidity and mortality associated with HIV-1 infection (Barre-Sinoussi et al., 2013). Although ART reduces plasma viral load below the limit of detection of current clinical tests (<40 copies HIV-RNA/ml plasma), viral rebound occurs in most HIV-infected individuals upon treatment interruption, including those receiving ART at a very early stage of primary infection (Ho and Zhang, 2000; Gianella et al., 2015; Henrich et al., 2017; Colby et al., 2018). The persistence of viral reservoirs during ART is well-established to represent a major barrier for HIV eradication (Chomont et al., 2011; Chun et al., 2015; Churchill et al., 2016; Deeks et al., 2016; Martin and Siliciano, 2016). Therefore, major efforts are invested toward the development of HIV cure/remission strategies (Margolis et al., 2016; Goulder and Deeks, 2018; Sengupta and Siliciano, 2018).

Replication-competent HIV reservoirs are established in long-lived memory CD4^+^ T-cells, with a frequency estimated as low as one infected cell *per* 1 × 10^6^ CD4^+^ T-cells (Finzi et al., 1997; Eisele and Siliciano, 2012; Massanella et al., 2018; Siliciano and Siliciano, 2018). Multiple groups, including ours, documented the fact that HIV-DNA reservoirs are enriched in CD4^+^ T-cells with unique phenotypes and functions, including central memory (T_*CM*_) (Chomont et al., 2009), stem cell memory (T_*SCM*_) (Buzon et al., 2014), helper (Th) type 17 (Th17) (Sun et al., 2015; Khoury et al., 2016; Gosselin et al., 2017; Planas et al., 2017, 2018; Anderson et al., 2019), Th1 (Lee et al., 2017), and/or follicular helper T-cells (Tfh) (Banga et al., 2016; Miles and Connick, 2016). Different HIV cure strategies have been proposed and/or are under pre-clinical evaluation, including “*shock and kill*” strategies using latency reversing agents (LRAs), gene therapy, and most recently “*block and lock*” strategies using Tat inhibitors (Buzon et al., 2010; Archin et al., 2012; Archin and Margolis, 2014; Chun et al., 2015; Mousseau et al., 2015a; Margolis et al., 2016; Kessing et al., 2017; Kim et al., 2017). To evaluate the efficacy of such strategies, progress has been made in the field of HIV reservoir quantification (Eriksson et al., 2013; Bruner et al., 2015, 2019; Massanella and Richman, 2016; Massanella et al., 2018). Despite these advances, the accurate quantification of clinically-relevant replication-competent HIV reservoirs using standardized assays remains a key research priority for orienting treatment interruption upon HIV curative interventions.

To quantify HIV reservoirs, the assays developed to date include PCR/RT-PCR-based and cell-culture based approaches (Bruner et al., 2015; Massanella and Richman, 2016; Massanella et al., 2018; Siliciano and Siliciano, 2018; Wang et al., 2018). Among PCR-based assays, the quantification of integrated HIV-DNA using HIV/Alu primers and FRET-based nested real-time PCR provides an estimate of the frequency of cells carrying HIV proviruses (Brussel and Sonigo, 2003; Chomont et al., 2009; Pinzone and O’Doherty, 2018). Although this method allows a simple, scalable, and low cost estimation of viral reservoir size, a large proportion of integrated HIV-DNA is defective (Eriksson et al., 2013; Ho et al., 2013; Cohn et al., 2015; Bruner et al., 2016; Deeks et al., 2016; Kiselinova et al., 2016; Lorenzi et al., 2016); therefore, such approaches largely over-estimate the size of replication-competent HIV reservoirs (Bruner et al., 2015; Deeks et al., 2016). Most recently, a multiplex PCR-based approach, entitled intact proviral DNA assay (IPDA), was established to quantify intact versus defective HIV proviruses (Bruner et al., 2019).

To quantify transcriptionally-competent HIV proviruses, one approach is to measure levels of cell-associated HIV-RNA upon short-term activation *in vitro*, where the fractional provirus expression (fPVE) upon TCR triggering and/or LRAs stimulation is measured (Cillo et al., 2014). To this aim, the Tat/Rev Induced Limiting Dilution Assay (TILDA) was developed to quantify the frequency of CD4^+^ T-cells expressing multiply spliced HIV-RNA upon short-term PMA/Ionomycin stimulation *in vitro* (Procopio et al., 2015). One step further in the quantification of HIV reservoirs at a single-cell level is now offered by HIV-Flow (Pardons et al., 2019) and the flow cytometry-based intracellular staining and *in situ* hybridization assay (Flow-FISH) that quantifies transcription/translation-competent HIV reservoirs *via* the detection of cells co-expressing HIV-RNA and the HIV capsid protein (HIV-p24) (Baxter et al., 2016, 2017, 2018). Similar to the PCR methods, the fPVE, TILDA, HIV-Flow, and Flow-FISH assays also overestimate the size of HIV reservoirs considering the fact that not all transcription/translation events lead to the production of infectious virions.

Quantitative viral outgrowth assays (QVOAs) estimate the frequency of resting CD4^+^ T-cells harboring replication-competent proviruses in the peripheral blood of ART-treated individuals (Finzi et al., 1997; Eriksson et al., 2013; Bullen et al., 2014; Bruner et al., 2015; Martin and Siliciano, 2016). The frequency of such reservoirs is significantly lower compared to the frequency of cells carrying integrated HIV-DNA, in line with findings demonstrating that a large proportion of proviruses is defective (Wong et al., 1997; Eriksson et al., 2013; Ho et al., 2013; Cohn et al., 2015; Bruner et al., 2016; Deeks et al., 2016; Kiselinova et al., 2016; Lorenzi et al., 2016; Siliciano and Siliciano, 2018). Classical QVOAs are labor intensive and time consuming, requiring multiple replicates in serial dilution and co-culture with irradiated PBMCs as feeder cells and/or CD8-depleted lymphoblasts from uninfected individuals as target cells for efficient amplification of replication-competent virions (Siliciano and Siliciano, 2005; Bruner et al., 2015; Laird et al., 2016; Massanella and Richman, 2016). Simplified versions of QVOAs use the indicator HIV-permissive cell lines MOLT-4/CCR5 (Laird et al., 2013) or Sup T1 CCR5^+^ (Fun et al., 2017). The sensitivity of the QVOA was improved by introducing the RT-PCR measurement of viral RNA (Laird et al., 2013) instead of the measurement of HIV-p24 in cell-culture supernatants by ELISA (Finzi et al., 1997). Despite these improvements, the sensitivity of QVOAs remains suboptimal, as reflected by the inability to detect HIV outgrowth in all tested samples even by using large numbers of CD4^+^ T-cells in multiple replicates (Laird et al., 2013; Siliciano and Siliciano, 2018). Indeed, several rounds of reactivation are needed to reverse latency in specific CD4^+^ T-cell subsets (Laird et al., 2013; Bruner et al., 2015; Hosmane et al., 2017; Siliciano and Siliciano, 2018; Wang et al., 2018), indicative that current cell activation strategies are suboptimal for efficient viral reactivation/outgrowth. These are critical limitations, especially for studies on small blood samples (e.g., pediatric samples), as well as cells isolated from deep tissue upon biopsy and/or autopsy.

In this manuscript, we provide new insights into the optimization of a simplified viral outgrowth procedure (VOP), in which memory CD4^+^ T-cells of ART-treated PLWH were stimulated *via* the TCR, cultured in the presence of *all-trans* retinoic acid (ATRA), and maintained at optimal density by regular splitting into new wells. Noteworthy, the viral outgrowth was robustly detected in the absence of feeder/target cells or indicator cell lines upon 12 days of culture. Finally, the culture of the cells in four original replicates of 1 × 10^6^ cells/well in 48-well plates and six original replicates of 2 × 10^5^ cells/well in 96-well plates allowed the calculation of the number of infectious units per million (IUPM) (Rosenbloom et al., 2015), with the detection of IUPMs being significantly increased by ATRA. These insights emerged from our experience in reactivating HIV reservoirs in rare subsets of CCR6^+^ Th17 cells (Wacleche et al., 2016), as well as the evidence generated by our group (Monteiro et al., 2011; Gosselin et al., 2017; Planas et al., 2017) and others (Cicala et al., 2009; Martinelli et al., 2011; Li et al., 2016) that ATRA increases HIV permissiveness *in vitro* and viral outgrowth *ex vivo*. In conclusion, cell-culture strategies presented in this manuscript are important to simplify current QVOAs for scalable viral reservoir quantifications in clinical trials using small biological samples.

## Materials and Methods

### Subjects

HIV-infected study participants were recruited at the McGill University Health Centre and at the Centre Hospitalier de l’Université de Montréal (CHUM, Montreal, Québec, Canada). Large quantities of peripheral blood mononuclear cells (PBMCs) (10^9^–10^10^) were collected by leukapheresis (Boulassel et al., 2003) and cryopreserved until use, as previously reported (Chomont et al., 2009; Wacleche et al., 2016; Gosselin et al., 2017; Planas et al., 2017). The clinical characteristics of HIV-infected study participants treated or not with ART at the time of blood collection are listed in Supplementary Table S1.

### Ethics Statement

This study, using PBMC samples from HIV-infected subjects, was conducted in compliance with the principles included in the Declaration of Helsinki. This study received approval from the Institutional Review Board of the McGill University Health Centre and the CHUM-Research Centre, Montreal, QC, Canada. Written informed consents and agreement to publish the results were obtained from all study participants.

### Flow-Cytometry Analysis

Surface staining for flow cytometry analysis was performed with the following fluorochrome-conjugated antibodies (Abs): CD3 Pacific blue (clone UCHT1), CD4 Alexa700 (clone RPA-T4; BD Biosciences), and CD45RA APC-eFluor 780 (clone HI100; eBioscience). Intracellular staining was performed with FITC-conjugated HIV-p24 Abs (clone KC57; Beckman Coulter) and the Fixation/Permeabilization Kit (BD). The LIVE/DEAD Fixable Aqua Dead Cell Stain Kit (Invitrogen) was used to exclude dead cells. Flow-cytometry analysis was performed using a LSRII cytometer, Diva version 8 (BD Biosciences, San Jose, CA, United States), and FlowJo version 10.0.6 (Tree Star, Inc.). Flow cytometry gates were defined using the *fluorescence minus one* (FMO) strategy (Roederer, 2002; Gosselin et al., 2010).

### Cell Sorting

Highly pure (>95%) total memory (CD45RA^–^) CD4^+^ T-cells were enriched from PBMCs by negative selection using magnetic beads (MACS, Miltenyi) (Monteiro et al., 2011; Gosselin et al., 2017). Memory CCR6^+^ and CCR6^–^ CD4^+^ T-cells were further isolated by flow cytometry (BD Aria III), using specific procedures and antibodies previously described (Planas et al., 2017).

### Quantification of Integrated HIV-DNA

The quantification of integrated HIV-DNA levels was performed in triplicates on 0.5–1 × 10^5^ CD4 T-cells by nested real-time PCR, as we previously described (Chomont et al., 2009; Gosselin et al., 2017). To ensure accurate integrated HIV-DNA quantification for non-clade B HIV, primers were designed to amplify viral genomes from a larger number of HIV clades (A, B, C, D, and CRF01A_E), as we previously described (Vandergeeten et al., 2014).

### Viral Outgrowth Procedure (VOP)

Memory CD4^+^ T-cells were cultured at 1 × 10^6^ cells/well in 1 ml of media (RPMI, 10% FBS, 1% Penicillin/Streptomycin) in a 48-well plate (Costar) coated with CD3 Abs (1 μg/ml, Clone UCHT1) and in the presence of soluble CD28 Abs (1 μg/ml) (BD Biosciences, Clone CD28.2). At day 3, cells from each original replicate were individually washed with media, split into two new CD3 Abs-uncoated 48-well plate wells, and cultured in media containing IL-2 (5 ng/ml; R&D Systems), in the presence or in the absence of ATRA (10, 100, 1,000, and 10,000 nM; SIGMA). The equivalent volume of DMSO was used as a control for ATRA. Cells from each well were further split into two new wells (without washing) at day 6 and 9 post-stimulation, with half of the media being refreshed with IL-2 with/without ATRA. Thus, each original replicate generated eight splitting replicates at days 9–12. At day 12, cells from all of the splitting replicates were individually harvested and the intracellular expression of HIV-p24 was measured by flow-cytometry, with the percentage of cells expressing intracellular HIV-p24 reflecting viral outgrowth in culture but not the size of the initial viral reservoir. In parallel, soluble HIV-p24 levels were quantified in cell-culture supernatants collected every three days post-stimulation using a previously described home-made ELISA assay (limit of detection: 5 pg/ml HIV-p24) (Bounou et al., 2002). ELISA optical density (OD) values for samples were considered positive only when they exceeded two times the OD value of the blank.

### ATRA-Based Quantitative Viral Outgrowth Assay (ATRA-QVOA)

To render the VOP quantitative, memory CD4^+^ T-cells isolated from ART-treated PLWH were cultured in multiple original replicates of 1 × 10^6^ cells/well in 48-well plates (four original replicates at day 0, generating 32 splitting replicates at days 9–12) and 2 × 10^5^ cells/well in 96-well plates (6 original replicates at day 0, generating 48 splitting replicates at days 9–12) using the VOP described above, as depicted in Figure 4A. Cultures were performed in the presence or in the absence of ATRA (100 nM). The control for ATRA was medium instead of DMSO; this simplification of the protocol for the VOP as described above was based on preliminary results demonstrating that an equivalent volume of DMSO (0.01 μl/ml) had no impact on the magnitude and the kinetics of HIV replication *in vitro* (data not shown). At day 12, HIV-p24 levels were measured by ELISA in cell-culture supernatants collected separately from all individual splitting replicates. For the calculation of IUPM one original replicate was considered positive if the HIV-p24 values were positive for at least two consecutive time points (typically days 9 and 12) and in one/multiple splitting replicates (Supplementary Figure S5). The IUPM values were calculated using the formula established by the group of Dr. Siliciano (https://silicianolab.johnshopkins.edu) (Rosenbloom et al., 2015).

### Statistical Analysis

Figures were generated using the Prism 7 (GraphPad) software. Specifications relative to the statistical tests used are included in the results and figure legends. Briefly, statistical analysis were performed using R [R Core Team (2017); R: A language and environment for statistical computing. R Foundation for Statistical Computing, Vienna, Austria (https://www.R-project.org/)]. We used R packages lme4 to fit the linear mixed regression (Supplementary Tables 3A,B, 4A,B) model and glmmTMB package to fit the negative binomial generalized regression model (Supplementary Tables S5A,B). The aov() and pairwise t.test() functions implemented in the R package stats were used for the paired ANOVA analysis (Supplementary Tables S2A,B).

## Results

### A Simplified Viral Outgrowth Procedure Using Memory CD4^+^ T-Cells of ART-Treated PLWH

**FIGURE 1:**
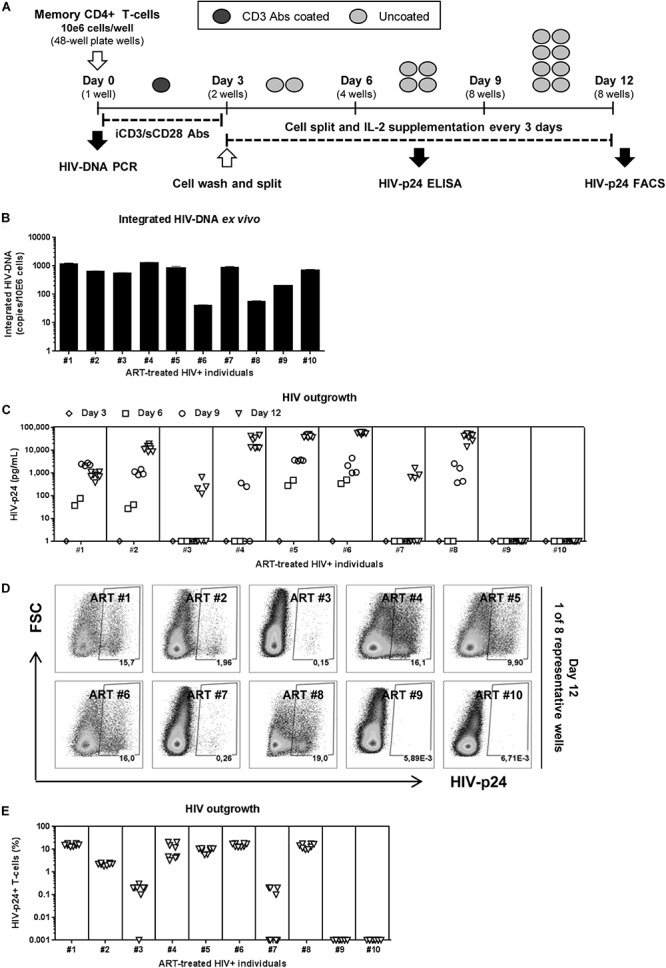
HIV reservoir outgrowth in memory CD4^+^ T-cells of HIV-infected ART-treated individuals. Memory CD4^+^ T-cells were isolated by MACS from PBMCs of ART-treated PLWH (HIV+ART) individuals (Supplementary Table S1; HIV+ART #1–10). **(A)** Shown is the viral outgrowth procedure (VOP) performed as described in the Results and Materials and Methods. **(B)** Shown are integrated HIV-DNA levels (mean ± SD of triplicate wells) in memory CD4^+^ T-cells (10^5^ cells/PCR reaction) from each donor *ex vivo*. **(C)** Shown are HIV-p24 levels measured by ELISA in cell-culture supernatants collected at day 3 (1 well/donor), day 6 (2 wells/donor), day 9 (4 wells/donor), day 12 (8 wells/donor) post-culture. At day 12 post-stimulation, cells were harvested and used for intracellular staining with HIV-p24 Abs and subsequent flow cytometry analysis. Shown are **(D)** representative flow cytometry dot plot and **(E)** graphical representation of the percentage of HIV-p24^+^ T-cells (8 wells/individual) in *n* = 10 HIV+ART individuals. Each symbol represents one experimental replicate well, with a total of 8 wells/individual at day 12.

Our goal was to optimize a new simplified VOP suitable for relatively small blood volumes collected from ART-treated PLWH. This VOP was performed in the absence of allergenic feeder cells, lymphoblast target cells, or HIV-permissive indicator cell lines. This VOP is based on our previous reports demonstrating successful HIV outgrowth from as low as 0.5 × 10^6^ flow-cytometry-sorted memory CCR6^+^ Th17 subsets from ART-treated PLWH (Wacleche et al., 2016). Briefly, one original replicate of 1 × 10^6^ memory CD4^+^ T-cells/well were cultured in 48-well plates coated with CD3 Abs (iCD3) and cultured in the presence of soluble CD28 (sCD28) Abs (1 μg/ml), a T-cell activation strategy that proved to be optimal for promoting HIV outgrowth in other studies (Spina et al., 2013; Cillo et al., 2014; Beliakova-Bethell et al., 2017). Of note, although increased T-cell activation (i.e., CD25 expression) and proliferative potential (i.e., Ki67 expression) were observed by increasing the concentration of CD3/CD28 Abs (>1 μg/ml) or upon activation with CD3/CD28-coated Dynal beads, unexpectedly, this strong T-cell activation led to decreased HIV outgrowth; this result is indicative that a low/moderate TCR triggering is required for optimal HIV outgrowth (data not shown). The VOP was performed as illustrated in Figure 1A and detailed in section Materials and Methods.

To test the efficacy of this VOP, a first set of experiments was performed with memory CD4^+^ T-cells from five untreated PLWH with detectable plasma viral load (Supplementary Table S1; HIV+ #14–18). Regardless of inter individual differences in integrated HIV-DNA levels (Supplementary Figure S1A), robust HIV outgrowth from 1 × 10^6^ memory CD4^+^ T cells was detected in all 5/5 donors tested, as early as at day 6 post-stimulation (Supplementary Figures S1B,C). Thus, this VOP allowed the detection of replication-competent HIV in as low as 1 × 10^6^ memory CD4^+^ T-cells from 5/5 ART-untreated PLWH tested.

Further, the same VOP was performed using memory CD4^+^ T-cells from a cohort of virally-suppressed ART-treated PLWH (HIV+ART), with clinical parameters listed in Supplementary Table S1. Although relatively high levels of integrated HIV-DNA were detected in memory CD4^+^ T-cells from all 10/10 HIV+ART individuals (Figure 1B), HIV-p24 levels in the cell-culture supernatant were only detected by ELISA in cells from 8/10 individuals (Figure 1C). Similarly, the presence of HIV-p24^+^ T-cells at various frequencies was detected by flow-cytometry in cells from the same 8/10 individuals at day 12 post-stimulation (Figures 1D,E), indicative of efficient cell-to-cell spread of virions *in vitro*. Of note, viral outgrowth was undetectable in T-cells from donors ART#9 and ART#10 (Figures 1C–E), despite relatively high levels of integrated HIV-DNA detected in these donors (Figure 1B). These findings are consistent with the knowledge that replication-incompetent HIV proviruses exist in ART-treated individuals (Eisele and Siliciano, 2012; Eriksson et al., 2013; Ho et al., 2013; Bruner et al., 2016). Thus, this VOP allowed the successful detection of replication-competent HIV reservoirs in 80% of HIV+ART individuals tested using one original replicate of 1 × 10^6^ memory CD4^+^ T-cells and cell-culture for 12 days.

### Optimal Cell Density in Culture Is Key for HIV Outgrowth

**FIGURE 2:**
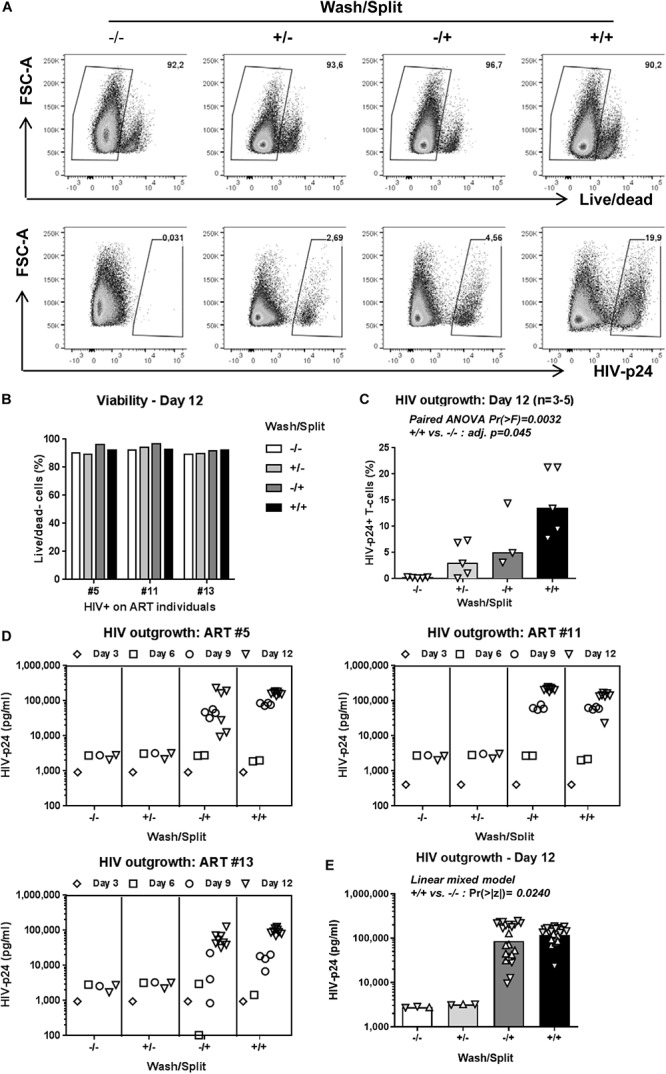
Optimal cell density in culture is key for HIV outgrowth. The VOP was performed as in Figure 1A using memory CD4^+^ T-cells from *n* = 3–5 HIV-infected ART-treated individuals. To determine the importance of washing at day 3 and splitting at days 3, 6 and 9 post-culture, the VOP was performed in parallel with cells that were washed and split (+/+), only washed (+/−) or split (−/+), and in the absence of (−/−). A viability dye was used to measure cell survival. Intracellular HIV-p24 expression was measured by flow-cytometry at day 12 of culture. Cells from eight wells at day 12 for the conditions with cell splitting (−/+ and +/+) were merged to generated one single value, as for the conditions without cell splitting (−/− and +/−). Shown are results on intracellular HIV-p24 levels and viability from one representative individual (ART #13) at day 12 **(A)**, as well as cell viability **(B)** and the percentage of HIV-p24^+^ T-cells at day 12 for *n* = 3–5 HIV+ART individuals **(C)**. Bars in **3C** are median values, with symbols for each HIV+ART individual. Paired ANOVA Pr(> F) value and Bonferroni *Post hoc* tests following pairwise paired *t*-tests adjusted (adj.) *p*-values for the + / + versus −/− comparison are indicated on the graph (details in Supplementary Table S2). Shown are HIV-p24 levels in cell culture supernatants at days 3, 6, 9, and 12 for three different HIV+ART individuals **(D)**. Bars in **3E** are geometric mean values, with distinct symbols for different wells/donor at Day 12 **(E)**. Linear mixed model Pr(>|z|) values for the +/+ versus −/− comparison are indicated on the graph (details in Supplementary Table S3).

The decision to wash cells at day 3 post-TCR triggering and to split cells every 3 days is justified by our previous observations that Th17 viability, proliferation, and production of lineage-specific cytokines are well-maintained under such experimental settings in long-term culture *in vitro* (Wacleche et al., 2016). To determine whether washing/splitting was important for HIV outgrowth, we performed the VOP in the presence or absence of washing and/or splitting in n = 3 HIV+ART individuals (HIV+ART #5, #11, #13; Supplementary Table S1). The HIV outgrowth was measured by flow cytometry at day 12 post-culture (Figures 2A,C) and by ELISA at days 3, 6, 9, and 12 post-culture (Figures 2D,E). Results depicted in Figure 2C, as well as **paired ANOVA** and Bonferroni *Post hoc* tests following pairwise paired *t*-test analyses in Supplementary Table S2, demonstrate that in the absence of washing/splitting (−/−) viral outgrowth was low/undetectable; cell washing alone (+/−) or splitting alone (−/+) increased the % of HIV-p24^+^ T-cells in specific donors, without reaching statistical significance when compared to the −/− condition. Noteworthy, a significant increase in HIV outgrowth was observed when cells were both washed and split (+/+) compared to the −/− condition (Figure 2C and Supplementary Table S2). Similar results were obtained when soluble HIV-p24 levels were measured in cell-culture supernatants, with **linear mixed model** analysis indicating a statistically significant increase in HIV outgrowth in the +/+ versus −/− conditions at both Days 9 and 12 (Figures 2D,E and Supplementary Table S3). Thus, while splitting and washing alone increased HIV outgrowth in specific donors, optimal viral outgrowth was observed when both washing and splitting were performed. Based on these results, both wash and split were performed in the subsequent experiments.

### Reproducibility of the New VOP

Another important issue for this new VOP was to determine its reproducibility. For this, the VOP was performed using memory CD4^+^ T-cells of HIV+ART individuals (HIV+ART #1, #5, and #7; Supplementary Table S1) in two independent experiments. Results in Supplementary Figure S2 demonstrate that this VOA is reproducible.

### ATRA Improves HIV Outgrowth in Memory CD4^+^ T-Cells

To improve the efficacy of this VOP, memory CD4^+^ T-cells of HIV+ART individuals were cultured in the presence or in the absence of ATRA, a stimulus demonstrated by our group and others to increase HIV replication either directly by increasing HIV transcription and/or indirectly by rendering neighboring cells highly permissive to infection (Cicala et al., 2009; Monteiro et al., 2011; Li et al., 2016; Gosselin et al., 2017). In a preliminary experiment, the VOP was performed with cells from HIV+ART#5, as described in Figure 1A, in the presence of different concentration of ATRA (10, 100, 1,000, and 10,000 nM). HIV-p24 levels were monitored in cell-culture supernatants by ELISA up to 12 days post-stimulation. Results in Supplementary Figure S3 demonstrate that ATRA significantly improved HIV outgrowth, especially at day 6 post-stimulation, with optimal HIV outgrowth being achieved with ATRA at a concentration of 100 nM.

**FIGURE 3:**
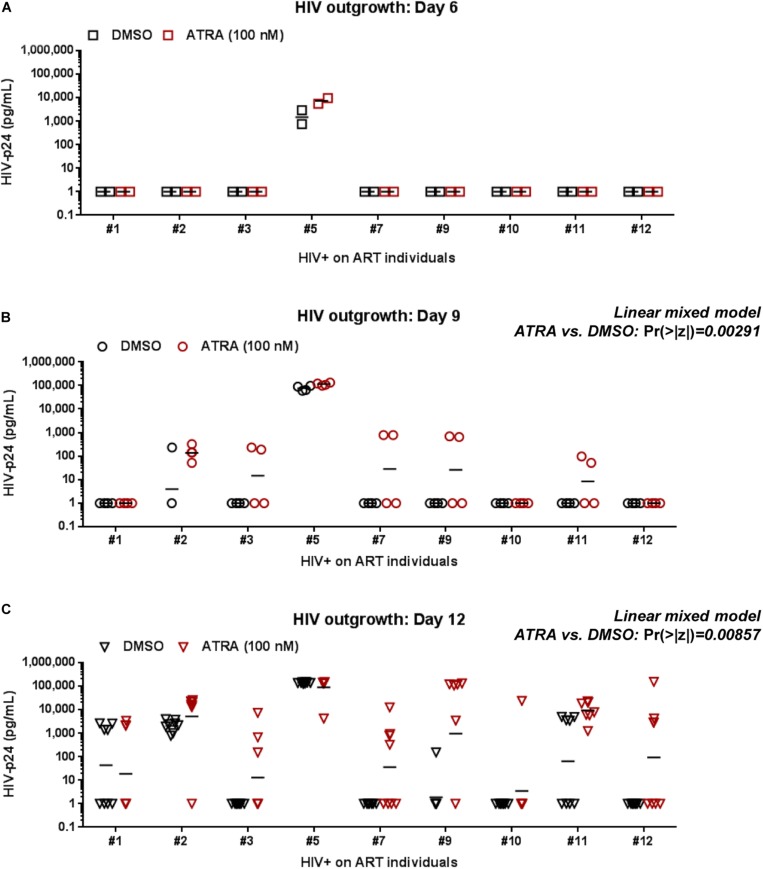
ATRA boosts HIV outgrowth. The VOP was performed with memory CD4^+^ T-cells of ART-treated PLWH (*n* = 9 HIV+ART individuals; Supplementary Table S1) in the presence (red symbols) or absence (black symbols) of ATRA (100 nM), as described in Figure 1. Shown are absolute HIV-p24 levels quantified in cell-culture supernatants collected from splitting replicates at days 6 (2 wells) **(A)**, 9 (4 wells) **(B)**, and 12 (8 wells) **(C)** post-culture. The horizontal black lines indicate the geometric means in **(A–C)**. Linear mixed model Pr (>|z|) values are indicated on the graphs for the comparison ATRA versus DMSO at Day 9 and Day 12 (details in Supplementary Table S4).

Further, the VOP was performed in the presence or the absence of 100 nM ATRA in memory CD4^+^ T-cells from *n* = 9 HIV+ART individuals (HIV+ART #1, #2, #3, #5, #7, #9, #10, #11, and #12; Supplementary Table S1). First we observed that ATRA allowed HIV outgrowth detection in cells from individuals where viral outgrowth was low/undetectable in the absence of ATRA, notably ART #3, #7, #9, and #10 (Figure 3C and Supplementary Figure S4). To determine the effect of ATRA on HIV outgrowth, we used a linear mixed model to compare HIV-p24 levels in supernatants form cell cultures performed in the presence or the absence of ATRA. Results in Figures 3B,C and Supplementary Table S4 demonstrate a statistically significant increase in HIV outgrowth per well at Day 9 (4 wells/donor; Pr (>|z|) = 0.00291) and Day 12 (8 wells per donor; Pr (>|z|) = 0.00857) in the presence of ATRA versus DMSO. Finally, by applying a negative binomial regression model, we observed a statistically significant increase in the number of HIV-p24 positive wells/donor in the presence of ATRA versus DMSO at Day 9 (Pr (>|z|) = 0.0232), with the difference being only marginally significant at Day 12 (Pr (>|z|) = 0.07076) (Supplementary Table S5). Together, these results are indicative of an improved HIV outgrowth in the presence of ATRA, both in terms of amplitude of the HIV outgrowth per well and the number of positive wells/donor and time point. Such improvements are important for the accurate quantification of the replication-competent HIV reservoir size.

### The ATRA-Based QVOA

**FIGURE 4:**
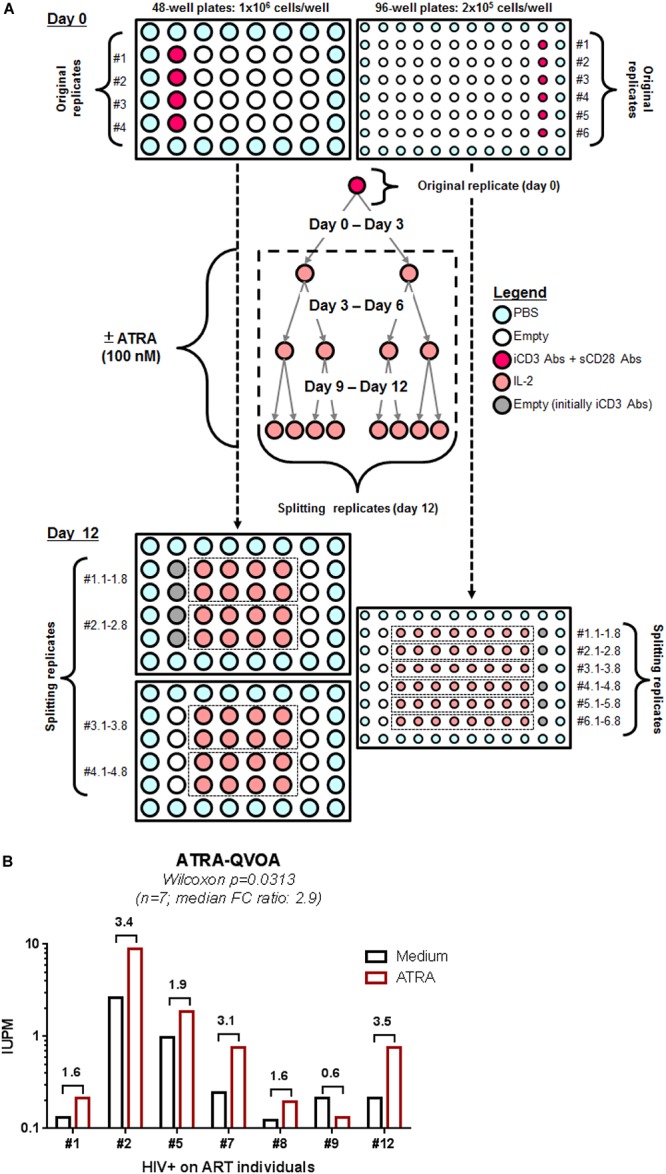
A simplified ATRA-based QVOA. The VOP was performed as described in Figure 1A legend, illustrated in Supplementary Figure S5, and described in section Materials and Methods using total memory CD4^+^ T-cells isolated from ART-treated PLWH. **(A)** Briefly, cells were cultured at two cell concentrations, 1 × 10^6^ cells/well in 48-well plates (4 original replicates) and 2 × 10^5^ cells/well in 96 well plates (6 original replicates) and stimulated via CD3/CD28 in the presence or absence of ATRA for 12 days. **(B)** Shown are the IUPM in cells from *n* = 7 HIV+ART PLWH (Supplementary Table S1; HIV+ART #1, #2, #5, #7, #8, #9, #12), when the QVOA was performed in the presence and in the absence of ATRA. Fold changes in IUPMs observed in the presence of ATRA versus Medium for each donor cells is annotated on the graph. Wilcoxon matched-pairs signed rank test *p*-value is indicated on the graph.

To render quantitative the VOP described above, four original replicates of 1 × 10^6^ cells/well (generating 32 splitting replicates at day 12) and six original replicates of 2 × 10^5^ cells/well (generating 48 splitting replicates at day 12) were cultured in 48- and 96-well plates, respectively, in the presence or in the absence of ATRA using a cell-culture protocol illustrated in Figure 4A. HIV-p24 quantification by ELISA of all individual splitting replicates allowed the calculation of the IUPM, using the formula established by the group of Dr. Robert Siliciano (https://silicianolab.johnshopkins.edu) (Rosenbloom et al., 2015). An original replicate was considered positive if one or more splitting replicates were positive at two consecutive time points for HIV-p24, as depicted in Supplementary Figure S5. Experiments performed with memory CD4^+^ T-cells from *n* = 7 HIV+ART individuals (Supplementary Table S1; HIV+ART #1, #2, #5, #7, #8, #9, and #12) demonstrated a statistically significant increase in the IUPM values when cells were cultured in the presence of ATRA (Figure 4B and Supplementary Figure S5B), with the IUPM geometric mean increasing from 0.3350 in the absence of ATRA to 0.6591 in the presence of ATRA (*n* = 7; *p* = 0.0313). In conclusion, the ATRA-based QVOA represents a simplified assay that requires a minimum of 5.2 × 10^6^ memory CD4^+^ T-cells, with the culture of cells in 4–6 replicates of two serial dilutions for 12 days allowing the proper calculation of the number of IUPMs.

Finally, we acknowledge that the HIV-p24 ELISA quantification of supernatants from all individual splitting replicates represents a heavy work load. However, the assay can be further simplified by merging supernatants from “identical” splitting replicates coming from one original well and also by testing only supernatants from days 9 and 12. Thus, instead of testing 150 individual supernatants (10 at day 3, 20 at day 6, 40 wells at day 9, and 80 wells at day 12) per donor, one may test only 20 merged “identical” supernatants (10 wells at day 9 and 10 wells at day 12). This strategy was tested in the laboratory and the IUPM calculation using this simplified strategy generated identical results (data not shown).

## Discussion

In this manuscript, we provide new insights into the optimization of the VOP from replication-competent HIV reservoirs by integrating tailored cell-culture strategies, including maintenance of optimal cell density by cell splitting, the preservation of all splitting replicates, as well as supplementing with ATRA, during 12 days in culture. This new VOP was performed with memory CD4^+^ T-cells from ART-treated PLWH in the absence of feeder/target cells or indicator cell lines. We demonstrate that culturing a minimum of 5.2 × 10^6^ memory CD4^+^ T-cells in 4 and 6 replicates of two serial dilutions for 12 days allows the calculation of IUPMs, with an efficacy that is significantly increased by ATRA. While this novel simplified ATRA-based QVOA is appropriate for HIV reservoir quantification in small biological samples, it may also serve as a platform to further improving the efficacy of QVOAs by supplementing the culture media with other stimuli that boost HIV reactivation in reservoir cells and facilitate cell-to-cell outgrowth in culture.

This VOP is built based on our expertise in reactivating HIV reservoirs from rare subsets of Th17 cells (Wacleche et al., 2016), a Th cell lineage that require suboptimal TCR triggering for the maintenance of effector functions, as well as culture at optimal cell density for access to nutrients (Kastirr et al., 2015; Mele et al., 2015). The detection of replication-competent HIV reservoirs in this VOP depends on multiple parameters, including the frequency of infected cells (HIV reservoir size), the capacity of reactivated virus to infect new cells (viral fitness), and also on the capacity of neighboring cells to support productive infection (HIV permissiveness). Cell washing at day 3 post-TCR triggering and cell splitting every 3 days significantly improved the efficiency of this VOP. One potential explanation for the importance of washing is that CD4^+^ T cells, particularly Th1 cells, produce CCR5 ligands (e.g., MIP-1β) that limit cell-to-cell transmission (Casazza et al., 2009; Gosselin et al., 2010; Alvarez et al., 2013). Since Th1 cells are predominant within the pool of CD4^+^ T-cells (Gosselin et al., 2010), the CCR5 ligands produced upon TCR triggering may limit the detection of viral outgrowth *in vitro*. Another explanation is that cytokines produced by Th1 cells (e.g., IFN-γ) are known to inhibit the transcriptional profile of Th17 cells (Basu et al., 2013; Wacleche et al., 2017), a subset enriched in HIV reservoirs (Gosselin et al., 2017; Planas et al., 2017, 2018; Anderson et al., 2019). Therefore, removal of such soluble factors from cell-culture supernatants remains a plausible explanation for viral outgrowth improvement. An additional improvement was achieved by splitting the cells, indicating that optimal cell density during the culture is critical for VOP, as reported for Th17 survival/function (Kastirr et al., 2015; Mele et al., 2015). The boost in HIV outgrowth by ATRA is explained by its documented capacity to promote HIV replication *via* entry (CCR5 upregulation) and post-entry mechanisms (mTOR activation) (Monteiro et al., 2011; Planas et al., 2017). Noteworthy, retinoic acid (RA) derivatives such as acitretin promote HIV transcription directly (Li et al., 2016), consistent with the presence of RA responsive elements, the DNA binding site of RA receptor alpha, within the HIV LTR (Lee et al., 1994).

Our effort was further focused on finding a strategy to render this ATRA-based VOP quantitative. Our results propose a simple strategy for calculating the IUPMs by culturing cells in multiple original replicates in only two serial dilutions and this for 12 days in culture. The sensitivity of the ATRA-based QVOA could be further improved by including more original replicates in the assay and by culturing the cells for more than 12 days. The positivity in this assay relies on the detection of HIV-p24 by ELISA in cell-culture supernatants. New ultrasensitive single molecule assays (SIMOA) have been recently developed for the detection of HIV-p24, with 1,000-fold superior sensitivity compared to classical HIV-p24 ELISAs (Fan et al., 2015; Kosaka et al., 2017; Passaes et al., 2017); whether SIMOA should be integrated to further optimize ATRA-based QVOAs remains to be determined. Nevertheless, in our ATRA-based QVOA, HIV-p24 levels at days 6–12 post-stimulation were robustly detected in cell-culture supernatants using classical ELISA assays. This new ATRA-based QVOA may be used successfully to quantify HIV reservoirs in small biological samples, but also for fundamental/translational purposes to test the effect of specific drugs acting positively/negatively on HIV transcription (Planas et al., 2017, 2018), a step untargeted by current ART (Barre-Sinoussi et al., 2013; Mousseau et al., 2015b). The IUPM calculation in this ATRA-QVOA can be further simplified by merging supernatants from identical splitting replicates at days 9 and 12 generated from one original well, thus significantly reducing the number of HIV-p24 ELISA wells per donor from 120 to 20. Such a simplification will also allow an increase in the number of original replicates, in an effort to reduce the confidence intervals for IUPM measures that proved to be relatively wide in the current ATRA-based QVOA.

We predict the performance of this ATRA-based QVOA may be further improved by performing the assay on sorted memory CCR6^+^ T-cells, documented to be enriched in HIV reservoirs (Gosselin et al., 2017; Planas et al., 2017, 2018; Anderson et al., 2019). An ATRA-based QVOA performed with memory CCR6^+^ T-cells may better orient treatment interruption decisions during HIV cure/remission therapeutic interventions in individuals with low/undetectable viral reservoirs detected in resting, total or memory CD4^+^ T-cells. Alternatively, the ATRA-based QVOA may be performed on other rare T-cell subsets reported to be enriched in viral reservoirs *in vivo*, such as Tfh (Banga et al., 2016) or PD1^+^ T-cells (Fromentin et al., 2019), in an effort toward the accurate quantification of intact replication-competent viral reservoirs.

The ATRA-based QVOA we are reporting here may represent an alternative to classical QVOAs that are labor intensive, expensive, and that require several rounds of activation for the accurate estimation of HIV reservoir size (Laird et al., 2013; Soriano-Sarabia et al., 2014; Bruner et al., 2015, 2019; Fun et al., 2017). In this sense, future comparative studies are needed to determine the relative efficacy of these two assays. Noteworthy, a very recent study demonstrated an improved detection of replication-competent HIV reservoirs using a strategy to promote effector memory differentiation thus, emphasizing the importance of appropriate cell activation and culturing conditions for efficient HIV outgrowth quantification (Wonderlich et al., 2019). This new assay called differentiation QVOA (dQVOA) proved to be more efficient compared to the classical QVOA in detecting replication-competent HIV reservoirs (Wonderlich et al., 2019). Future studies are also needed to compare the ATRA-based QVOA and dQVOA in terms of efficacy and simplicity for scalable applications.

## Conclusion

In conclusion, by combining optimal memory CD4^+^ T-cell stimulation *via* the TCR and cell density in culture, together with knowledge on the ability of ATRA to promote HIV reactivation and cell-to-cell transmission in culture, we propose a new ATRA-based QVOA that is relatively simple and highly sensitive for the quantification of replication-competent HIV reservoirs in ART-treated PLWH. The fact that viral outgrowth was boosted by ATRA, a key modulator of immunity *via* the imprinting for gut-homing and the modulation of T-cell effector functions (Raverdeau and Mills, 2014; Erkelens and Mebius, 2017), points to its potential use for the optimization of current QVOAs. The existence of other molecular boosters of HIV outgrowth is not excluded and requires future investigations. Such optimizations should also consider current advances in understanding the HIV integration landscape (Hughes and Coffin, 2016) and the unique transcriptional programming of distinct CD4^+^ T-cell subsets (Ansel et al., 2006; Gaffen et al., 2014; Kallies and Good-Jacobson, 2017) that predominantly carry HIV reservoirs (e.g., Th17 versus Th1) (Sun et al., 2015; Wacleche et al., 2016; Gosselin et al., 2017; Lee et al., 2017; Planas et al., 2017).

## Data Availability Statement

All datasets generated for this study are included in the article/Supplementary  Material.

## Ethics Statement

The studies involving human participants were reviewed and approved by McGill University Health Centre and the CHUM-Research Centre, Montreal, QC, Canada. The patients/participants provided their written informed consent to participate in this study.

## Author Contributions

YZ designed experiments, generated results included in Figures 1–3, prepared figures, and wrote the manuscript. DP generated results in Figures 2, 4, prepared figures, and participated in manuscript writing. LR generated results in Figure 4, prepared figures, and participated in manuscript writing. MM contributed to HIV-DNA PCR quantifications and QVOA set-up. HC performed HIV-p24 ELISA and flow-cytometry quantifications. VW contributed to the initial experimental design. J-PG prepared heat maps. AG performed cell sorting and HIV-DNA PCR quantifications. MF and JP-R insured study participant recruitment, blood cell collection through leukapheresis, and/or access to clinical information. NC contributed with expertise, protocols, and reagents, as well as data analysis for QVOA. PA designed experiments, prepared figures, and wrote the manuscript. All authors revised and approved the manuscript.

## Conflict of Interest

J-PR performed contract research and/or served on Advisory Boards for Gilead Sciences Canada Inc., Merck Canada Inc., Abbvie Corp., ViiV Healthcare, Bristol Myers Squibb, Janssen Inc., Argos Pharmaceuticals from InnaVirVax, and Theravectys. PA’s laboratory receives research funding from Glaxo Smith Klein/NeoMed for projects different from the present study. PA serves as a Consultant at Merck Canada Inc. NC received research funding from EMD Serono. MF was employed by Alethia Biotherapeutics. J-PG was employed by Caprion. The remaining authors declare that the research was conducted in the absence of any commercial or financial relationships that could be construed as a potential conflict of interest. The reviewer ERW declared a shared affiliation, with no collaboration, with one of the authors, NC, to the handling editor at time of review.
