# Supplementary material for: Improving HIV Outgrowth by Optimizing Cell-Culture Conditions and Supplementing With all-trans Retinoic Acid
Source: Front Microbiol. 2020 May 15;11:902. doi: 10.3389/fmicb.2020.00902 (PMC7243435; doi:10.3389/fmicb.2020.00902)
Supplement: Supplementary file 1 [file Data_Sheet_1.PDF]

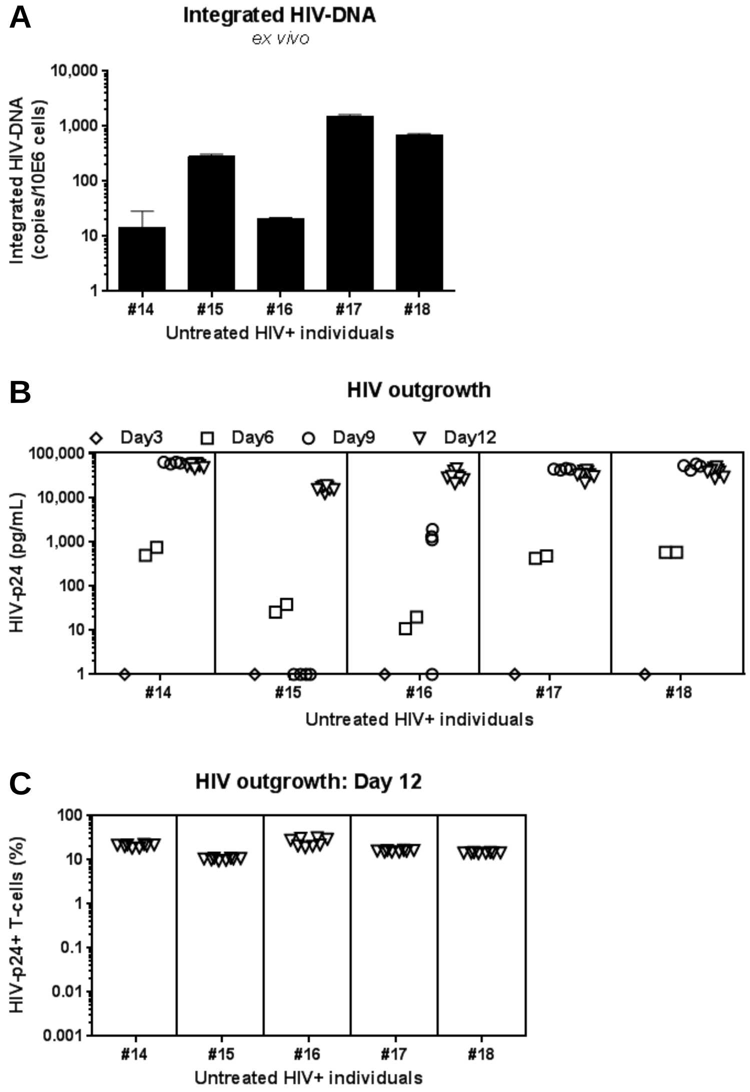

Zhang et al., Suppl. Figure 1

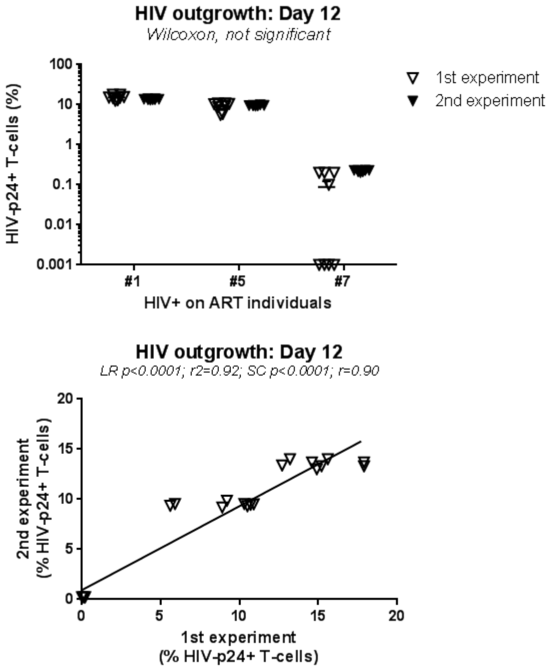

Zhang et al., Suppl. Figure 2

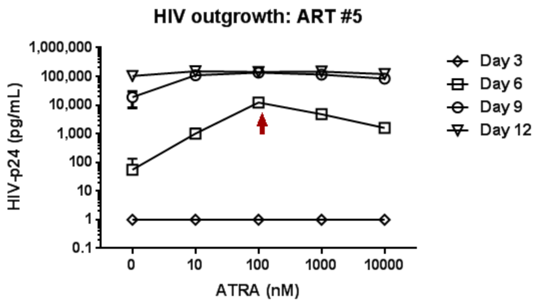

Zhang et al., Suppl. Figure 3

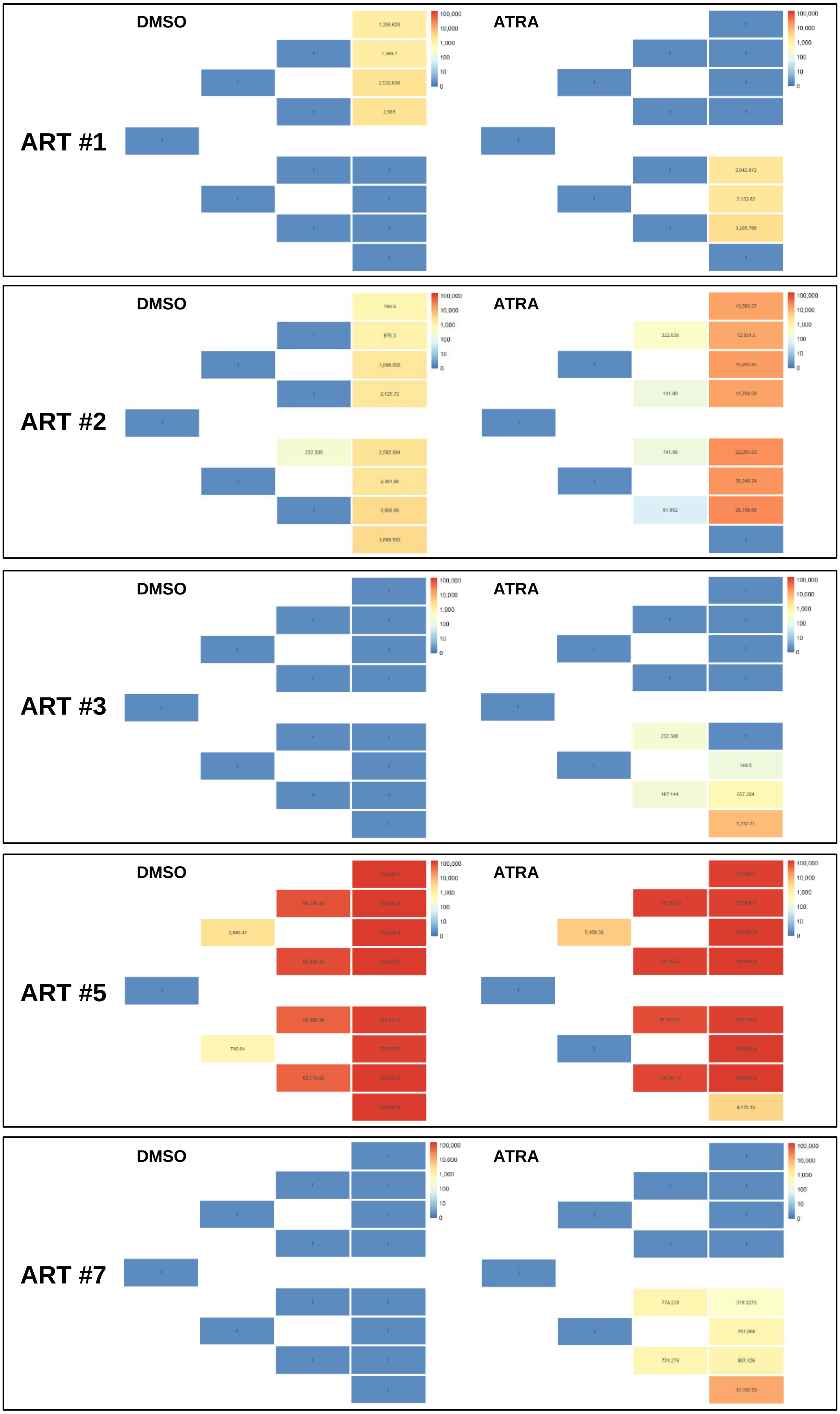

Zhang et al., Suppl. Figure 4

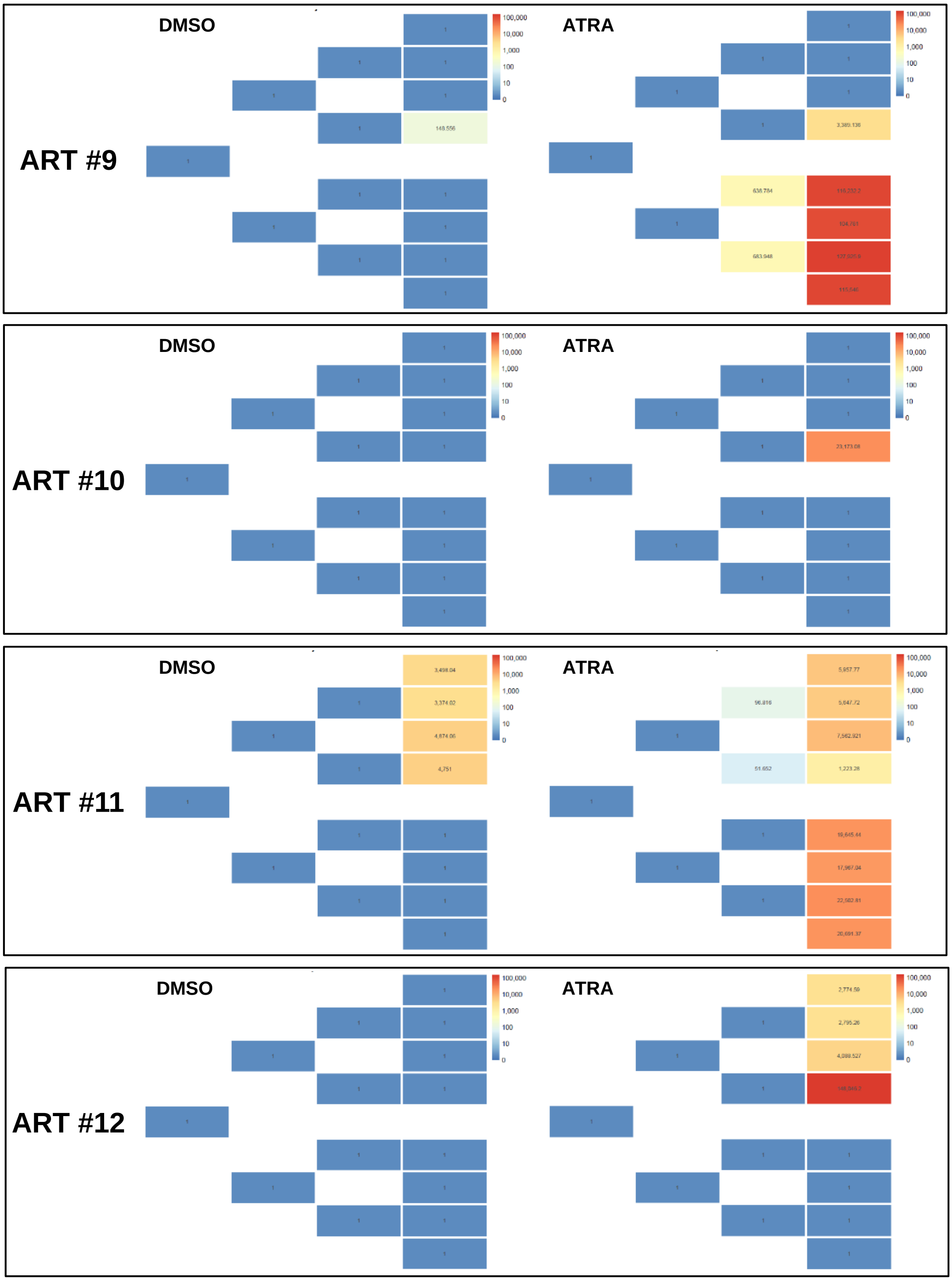

Zhang et al., Suppl. Figure 4 - continued

A. HIV-p24 ELISA quantification – HIV+ART #12

Medium — 48-well plate: 1x10[6] cells/well

Original replicates	Splitting replicates (HIV-p24 [pg/ml]) Day 9	Splitting replicates (HIV-p24 [pg/ml]) Day 12	Positivity
1	0	0	NO
		0	
	0	0	
		0	
	0	0	
		0	
	0	0	
		0	
2	0	0	NO
		0	
	0	0	
		0	
	0	0	
		0	
	0	0	
		0	
3	0	0	NO
		0	
	0	0	
		0	
	0	0	
		0	
	0	0	
		0	
4	0	0	YES
		48 601	
	1 708	0	
		32 955	
	0	0	
		33 755	
	2 576	0	
		34 628	

Medium — 96-well plate: 2x10[5] cells/well

Original replicates	Splitting replicates (HIV-p24 [pg/ml]) Day 9	Splitting replicates (HIV-p24 [pg/ml]) Day 12	Positivity
1	0	0	NO
		0	
	0	0	
		0	
	0	0	
		137	
	0	0	
		0	
2	0	0	NO
		0	
	22	0	
		0	
	0	0	
		0	
	0	0	
		0	
3	0	0	NO
		0	
	0	0	
		0	
	0	0	
		0	
	0	0	
		0	
4	0	0	NO
		0	
	0	0	
		0	
	0	0	
		0	
	21	0	
		0	
5	0	0	NO
		0	
	0	0	
		0	
	0	0	
		0	
	0	0	
		0	
6	0	0	NO
		0	
	0	0	
		0	
	0	0	
		0	
	0	0	
		0	

ATRA — 48-well plate: 1x10[6] cells/well

Original replicates	Splitting replicates (HIV-p24 [pg/ml]) Day 9	Splitting replicates (HIV-p24 [pg/ml]) Day 12	Positivity
1	0	0	NO
		0	
	0	13 524	
		0	
	0	0	
		0	
	0	10 648	
		0	
2	432	21 983	YES
		0	
	0	9 896	
		0	
	253	12 565	
		0	
	0	7 577	
		0	
3	0	0	NO
		0	
	0	0	
		0	
	0	0	
		0	
	0	0	
		0	
4	0	0	YES
		18 780	
	1 421	0	
		12 222	
	0	0	
		10 990	
	4 053	0	
		7 304	

ATRA — 96-well plate: 2x10[5] cells/well

Original replicates	Splitting replicates (HIV-p24 [pg/ml]) Day 9	Splitting replicates (HIV-p24 [pg/ml]) Day 12	Positivity
1	0	0	NO
		0	
	0	0	
		0	
	0	0	
		0	
	0	0	
		0	
2	0	0	NO
		0	
	0	0	
		0	
	0	0	
		0	
	0	0	
		0	
3	0	0	NO
		0	
	31	0	
		0	
	0	0	
		0	
	0	0	
		0	
4	0	0	NO
		0	
	0	0	
		0	
	0	0	
		0	
	0	0	
		0	
5	0	0	NO
		0	
	0	0	
		0	
	0	0	
		0	
	0	0	
		0	
6	0	8 000	YES
		0	
	0	20 254	
		0	
	42	5 710	
		0	
	0	18 740	
		0	

B. IUPM calculations

		$1x10^6$ cells/well (48-well plate)		$2x10^5$ cells/well (96-well plate)			95% confidence interval	
ART#	ATRA	Total wells	Positive wells	Total wells	Positive wells	**IUPM**	lower bound	upper bound
1	NO	**4**	0	**6**	0	**0,133298**	*	*
1	YES	**4**	1	**6**	0	**0,213574**	0,029972	1,521894
2	NO	**4**	3	**6**	4	**2,616424**	1,147623	5,965091
2	YES	**4**	4	**6**	5	**8,969419**	3,320795	24,226273
5	NO	**3**	2	**6**	0	**0,980829**	0,231885	4,148714
5	YES	**3**	3	**6**	1	**1,856759**	0,626763	5,500567
7	NO	**3**	1	**8**	0	**0,245122**	0,034358	1,748771
7	YES	**4**	3	**8**	0	**0,767255**	0,240628	2,446435
8	NO	**4**	0	**8**	0	**0,123776**	*	*
8	YES	**4**	1	**8**	0	**0,196710**	0,027621	1,400933
9	NO	**4**	1	**6**	0	**0,213574**	0,029972	1,521894
9	YES	**4**	0	**6**	0	**0,133298**	*	*
12	NO	**4**	1	**6**	0	**0.213574**	0.029972	1.521894
12	YES	**4**	2	**6**	1	**0.755133**	0.239146	2.384426

ART#, ART-treated PLWH; ATRA, *all-trans* retinoic acid; IUPM, infectious units per million;
*, 95% confidence interval below 0,576
