# Supplementary material for: Improving HIV Outgrowth by Optimizing Cell-Culture Conditions and Supplementing With all-trans Retinoic Acid
Source: Front Microbiol. 2020 May 15;11:902. doi: 10.3389/fmicb.2020.00902 (PMC7243435; doi:10.3389/fmicb.2020.00902)
Supplement: Supplementary file 2 [file Data_Sheet_2.pdf]

Supplemental figure legends

Supplementary Figure 1: HIV outgrowth in CD4$^+$ T-cells of HIV-infected untreated PLWH. The VOP was performed as described in Figure 1A using memory CD4$^+$ T-cells from ART-untreated PLWH (n=5; Supplemental Table 1, HIV+#14-18). Shown are levels of integrated HIV-DNA (mean±SD of PCR triplicates) measured *ex vivo* **(A)**; as well as soluble HIV-p24 levels measured by ELISA at days 3 (1 replicate/donor), 6 (2 replicate/donor), 9 (4 replicate/donor), and 12 (8 replicate/donor) post-culture **(B)** and intracellular HIV-p24 levels measured by flow-cytometry at day 12 post-stimulation **(C)**. Each symbol represents one experimental splitting replicate, with a total of 8 splitting replicates/study participant at day 12.

Supplementary Figure 2: Reproducibility of the VOP. The VOP, performed as in Figure 1A, was repeated in two independent experiments using memory CD4$^+$ T-cells from three HIV+ART individuals (ART#1, ART#5, and ART#7; Table 1). Shown are **(A)** the frequencies of HIV-p24$^+$ T-cells at day 12 post-culture in the 1st (open triangles) and 2nd (filled triangles) experiment and **(B)** the correlation between the results generated in these two independent experiments calculated using SC (p and r values) and LR (p and r2 values) models**.** Each symbol represents one splitting replicate well/study participant.

Supplementary Figure 3: ATRA accelerates/increases HIV outgrowth in a dose-dependent manner. The VOP was performed as described in Figure 1A with memory CD4$^+$ T-cells from one study participant (HIV+ART #5; Supplemental Table 1) in the presence of different doses of ATRA (10, 100, 1,000, and 10,000 nM) or the equivalent

volumes of DMSO as control. Shown are levels of HIV-p24 in cell-culture supernatants collected at days 3, 6, 9 and 12 post-culture. The red arrow indicates the optimal ATRA concentration (100 nM) subsequently used in Figures 3-5.

Supplemental Figure 4: ATRA boosts HIV outgrowth. The VOP was performed as described in Figure 1A with memory $CD4^+$ T-cells from HIV+ART individuals (n=9; Supplemental Table 1) and in the presence or in the absence of ATRA (100 nM). Shown are heatmap representations of HIV-p24 levels in cell-culture supernatants collected at days 3, 6, 9, and 12 post-stimulation in all n=9 donors. The heatmaps reflect the magnitude of soluble HIV-p24 levels *per* well and *per* donor at different time points (red for the highest and blue for the lowest HIV-p24 levels as indicated in the scale). The absolute HIV-p24 levels are indicated on the heat-map cells for positive wells.

Supplemental Figure 5: Calculation of IUPMs in the ATRA-based QVOA. The QVOA was performed in the presence or in the absence of ATRA (100 nM), as depicted in Figure 4A. Shown are **(A)** HIV-p24 quantification results in the splitting replicates from one representative donor (HIV+ART #12) and **(B)** a summary of HIV-p24 positivity in the original wells and the mode of IUPM calculation for all donors.
