# Supplementary material for: Improving HIV Outgrowth by Optimizing Cell-Culture Conditions and Supplementing With all-trans Retinoic Acid
Source: Front Microbiol. 2020 May 15;11:902. doi: 10.3389/fmicb.2020.00902 (PMC7243435; doi:10.3389/fmicb.2020.00902)
Supplement: Supplementary file 3 [file Data_Sheet_3.doc]

**Supplemental Table 1:** Clinical parameters of HIV-infected study participants

| **Subject ID** | **CD4^#^** | **CD8^#^** | **CD4/CD8 ratio** | **Age//** | **VL^&^** | **Time since**  **infection*** | **Current ART regimen** | **Time on ART*** |
| --- | --- | --- | --- | --- | --- | --- | --- | --- |
| **HIV+ART #1** | 398 | 775 | 0.51 | 44 | <40 | 154 | Complera | 25 |
| **HIV+ART #2** | 542 | 803 | 0.67 | 36 | <40 | 13 | Stribild | 12 |
| **HIV+ART #3** | 743 | 899 | 0.83 | 52 | <40 | 171 | Truvada/Raltegravir | 99 |
| **HIV+ART #4** | 458 | 899 | 0.51 | 49 | <40 | 227 | Truvada/Viramune | 201 |
| **HIV+ART #5** | 841 | 1,322 | 0.64 | 51 | <40 | 149 | Sustiva/Truvada | 148 |
| **HIV+ART #6** | 598 | 605 | 0.99 | 30 | <40 | 80 | Stribild | 77 |
| **HIV+ART #7** | 425 | 1,156 | 0.37 | 47 | <40 | 182 | Atripla | 58 |
| **HIV+ART #8** | 908 | 854 | 1.06 | 33 | <40 | 89 | Stribild | 77 |
| **HIV+ART #9** | 963 | 644 | 1.50 | 55 | <40 | 123 | Prevista/Kivexa/Norvir | 107 |
| **HIV+ART #10** | 288 | 407 | 0.71 | 56 | <40 | 211 | Atripla | 207 |
| **HIV+ART #11** | 836 | 1,551 | 0.54 | 52 | <40 | 167 | Isentress / Tivicay | 161 |
| **HIV+ART #12** | 833 | 445 | 1.87 | 31 | <40 | 212 | Viracept/Truvada | 187 |
| **HIV+ART #13** | 796 | 399 | 0.41 | 21 | <40 | 8 | Stribild | 4 |
| **HIV+ART #14** | 581 | 1,060 | 0.55 | 47 | <40 | 98 | Sustiva/Truvada | 91 |
| **HIV+ #14** | 946 | 1,356 | 0.70 | 39 | 8,809 | 9 | untreated | N/A |
| **HIV+ #15** | 316 | 691 | 0.46 | 24 | 9,496 | 55 | untreated | N/A |
| **HIV+ #16** | 529 | 447 | 1.18 | 48 | 3,189 | 110 | untreated | N/A |
| **HIV+ #17** | 389 | 1,540 | 0.25 | 50 | 97,552 | 152 | untreated | N/A |
| **HIV+ #18** | 644 | 931 | 0.69 | 40 | 7,556 | 4 | untreated | N/A |

#, cells/µl; //, years; &, plasma viral load (VL, HIV-RNA copies/ml); *, months

**Supplemental Table 2:** **Flow cytometry analysis of HIV outgrowth in relationship with cell culture conditions.**

***Supplemental Table 2A – Day 12***

| **Paired ANOVA - % HIV+ T-cells** | | | | | |
| --- | --- | --- | --- | --- | --- |
|  | **Df** | **Sum Sq** | **Mean Sq** | **F** | **Pr(>F)** |
| **condition** | **3** | **568** | **189.4** | **9.123** | **0.0032**** |
| **residual** | **10** | **207.5** | **20.75** |  |  |

***Supplemental Table 2B – Day 12***

| **Bonferroni Post-hoc tests** **following** **pairwise paired t-tests** | | | |
| --- | --- | --- | --- |
|  | **-/-** | **-/+** | **+/-** |
| **-/+** | **1(0.18)** | **-** | **-** |
| **+/-** | **0.44(0.07)** | **1(0.65)** | **-** |
| **+/+** | **0.045(0.0075)** | **1(0.28)** | **0.218(0.036)** |

*Wash/split; Ajd. P-values (nominal p values)*

**Supplemental Table 3:** **ELISA analysis of HIV outgrowth in relationship with cell culture conditions.**

**Supplemental Table 3A – Day 9**

| **Linear mixed model - HIV-p24 concentration** | | | | |
| --- | --- | --- | --- | --- |
|  | **Estimate** | **Std. error** | **Z-value** | **Pr(>|z|)** |
| **Intercept** | **3.43759** | **0.48601** | **7.073** | **1.51e-12 ***** |
| **-/+ *vs.* -/-** | **0.68137** | **0.51253** | **1.329** | **0.1837** |
| **+/- *vs.* -/-** | **0.06888** | **0.60071** | **0.115** | **0.9087** |
| **+/+ *vs.* -/-** | **1.17945** | **0.51253** | **2.301** | **0.0214 *** |

*Wash/split*

**Supplemental Table 3B – Day 12**

| **Linear mixed model - HIV-p24 concentration** | | | | |
| --- | --- | --- | --- | --- |
|  | **Estimate** | **Std. error** | **Z-value** | **Pr(>|z|)** |
| **Intercept** | **3.44035** | **0.63271** | **5.437** | **5.4e-08 ***** |
| **-/+ *vs.* -/-** | **1.13888** | **0.64357** | **1.770** | **0.0768** |
| **+/- *vs.* -/-** | **0.06074** | **0.84773** | **0.072** | **0.9429** |
| **+/+ *vs.* -/-** | **1.45237** | **0.64357** | **2.257** | **0.0240*** |

*Wash/split*

**Supplemental Table 4: Effect of ATRA on HIV outgrowth.**

**Supplemental Table 4A - Day 9**

| **Linear mixed model - HIV-p24 concentration** | | | | |
| --- | --- | --- | --- | --- |
|  | **Estimate** | **Std. error** | **Z-value** | **Pr (>|z|)** |
| **Intercept** | 1.1538 | 0.3932 | 2.934 | 0.00334 |
| **ATRA *vs.* DMSO** | 0.5038 | 0.1692 | 2.977 | **0.00291**** |

**Supplemental Table 4B - Day 12**

| **Linear mixed model - HIV-p24 concentration** | | | | |
| --- | --- | --- | --- | --- |
|  | **Estimate** | **Std. error** | **Z-value** | **Pr (>|z|)** |
| **Intercept** | 1.7786 | 0.4501 | 4.951 | 7.78e-05 *** |
| **ATRA *vs.* DMSO** | 0.7757 | 0.2951 | 2.629 | **0.00857 **** |

**Supplemental Table 5: Effect of ATRA on HIV outgrowth.**

**Supplemental Table 5A – Day 9**

| **Negative binomial regression model – number of positive/negative wells** | | | | |
| --- | --- | --- | --- | --- |
|  | **Estimate** | **Std. error** | **z-value** | **Pr (>|z|)** |
| **Intercept** | -0.95 | 0.58 | -1.6 | 0.1 |
| **ATRA *vs.* DMSO** | 1.1632 | 0.5123 | 2.270 | **0.0232*** |

**Supplemental Table 5B – Day 12**

| **Negative binomial regression model – number of positive/negative wells** | | | | |
| --- | --- | --- | --- | --- |
|  | **Estimate** | **Std. error** | **z-value** | **Pr (>|z|)** |
| **Intercept** | 0.83 | 0.3114 | 2.689 | 0.00716 |
| **ATRA *vs.* DMSO** | 0.5514 | 0.3051 | 1.807 | **0.07076** |
